# Vitamin intake and colorectal cancer incidence and mortality: evidence from a large prospective cohort

**DOI:** 10.3389/fnut.2026.1877860

**Published:** 2026-09-09

**Authors:** Le-Lan Gong, Qi-Wu, Geng-Xue, Feng-Chang Huang, Li Pu, Ning-Xu, Wen-Liang Li

**Affiliations:** 1 The Third Affiliated Hospital of Kunming Medical University, Kunming, Yunnan, China; 2 The Second Affiliated Hospital of Kunming Medical University, Kunming, Yunnan, China; 3 College of Food Science and Technology, and Yunnan Key Laboratory of Precision Nutrition and Personalized Food Manufacturing, Yunnan Agricultural University, Kunming, Yunnan, China; 4 Department of Oncology, the First Affiliated Hospital of Kunming Medical University, Kunming, Yunnan, China

**Keywords:** cancer prevention, colorectal cancer (CRC), prospective cohort, protective factor, vitamin

## Abstract

**Background:**

Emerging evidence suggests that vitamin intake may be associated with colorectal cancer (CRC) risk, yet prospective studies on CRC incidence and mortality remain limited. We therefore investigated the associations between vitamin intake and CRC incidence and mortality in a large cohort of US adults.

**Methods:**

We included 101,692 participants from the Prostate, Lung, Colorectal, and Ovarian Cancer Screening Trial. Vitamin intake (including dietary and supplement sources) was assessed using the Dietary History Questionnaire based on the USDA 1994–1996 survey and the University of Minnesota Nutrition Data System for Research. Prospective associations with CRC incidence and mortality were estimated using Cox proportional hazards regression models. Restricted cubic spline regression was applied to evaluate potential nonlinear relationships. Subgroup analyses were conducted to explore potential effect modifiers of CRC incidence and mortality.

**Results:**

During 896,655 person-years of follow-up (mean 8.82 years), 1,085 participants were diagnosed with CRC, and 529 CRC deaths occurred during 1,533,506 person-years of follow-up (mean 15.08 years). In fully adjusted models, higher intakes of vitamin A (HR = 0.75, 95% CI: 0.60–0.93; *P*-trend = 0.005), vitamin B6 (HR = 0.74, 95% CI: 0.61–0.90; *P*-trend = 0.015), vitamin D (HR = 0.77, 95% CI: 0.64–0.93; *P*-trend = 0.022), and vitamin E (HR = 0.81, 95% CI: 0.68–0.97; *P*-trend = 0.008) were significantly associated with a lower incidence of CRC. In addition, higher intakes of vitamins A, B6, and C were significantly associated with reduced CRC mortality. No significant associations were observed for vitamin B12 with CRC incidence and mortality. Sensitivity and subgroup analyses supported the robustness of these findings.

**Conclusion:**

In conclusion, higher intake of vitamins A, B6, C, D, and E is associated with an decreased risk of CRC incidence and mortality. Appropriate vitamin intake may represent a modifiable factor for the primary prevention of CRC.

## Introduction

Colorectal cancer (CRC) remains a major global public health challenge, with approximately 1,900,000 new cases and 935,000 related deaths reported worldwide in 2022 alone (1), ranking as the second leading cause of cancer-related mortality globally (2). The development of CRC is influenced by a complex interplay of genetic, environmental, and lifestyle factors, often accompanied by changes in the intestinal microenvironment (3). Dietary factors is one of the important factors in regulating the intestinal microenvironment, and Western dietary patterns may lead to CRC through mechanisms such as energy excess, micronutrient deficiencies, metabolic disorders, inflammation, oxidative stress, and dysbiosis of gut microbiota (4, 5). Dietary guidelines frequently emphasize single macro-nutrients in isolation without considering the impact of micronutrients. However, micronutrient intake is usually associated with dietary structure, and micronutrient deficiencies are still common in high-income countries where dietary patterns are energy-dense but nutrient-poor. In North American populations, vitamins are the most frequently used dietary supplements, with adults consuming at least 5 times as much vitamins B1, B2, and B6 from supplements than from food, and 15 to 20 times as much vitamins B12 and E from supplements (6). Therefore, modifying dietary habits, especially focusing on vitamin intake, should be a key aspect of primary prevention strategies for CRC.

Despite the limited research on vitamins and CRC, epidemiological studies have suggested an inverse association between vitamin intake and CRC incidence and mortality (7, 8). Several studies have linked vitamin D intake with a lower risk of early-onset CRC, indicating that higher total vitamin D intake can decrease this risk (7, 9, 10). However, a Danish cohort study involving 57,053 participants found no correlation between vitamin A intake and CRC development (11). The US Preventive Services Task Force has also indicated that the evidence is insufficient to determine the balance of benefits and harms of supplementation with single or paired nutrients for CRC. A randomized trial reported that modest benefits were observed for overall cancer incidence, with no association with cancer mortality, highlighting the lack of CRC-specific evidence (12). Therefore, a comprehensive evaluation of multiple vitamins in relation to CRC risk is warranted. In this large prospective cohort study, we systematically investigated the associations of multiple vitamin intakes with CRC incidence and mortality, thereby providing population-based evidence to inform CRC prevention strategies.

## Methods

Comprehensive details are available in the Supplementary materials.

### Study population

This cohort was drawn from the Prostate, Lung, Colorectal, and Ovarian (PLCO) Cancer Screening Trial, which enrolled about 155,000 individuals aged 55–74 years between 1993 and 2001. Participant characteristics and lifestyle information were collected through standardized questionnaires, including the Baseline Questionnaire (BQ) and the validated Diet History Questionnaire (DHQ), from which dietary nutrient and food group intakes were quantified.

**Figure 1:**
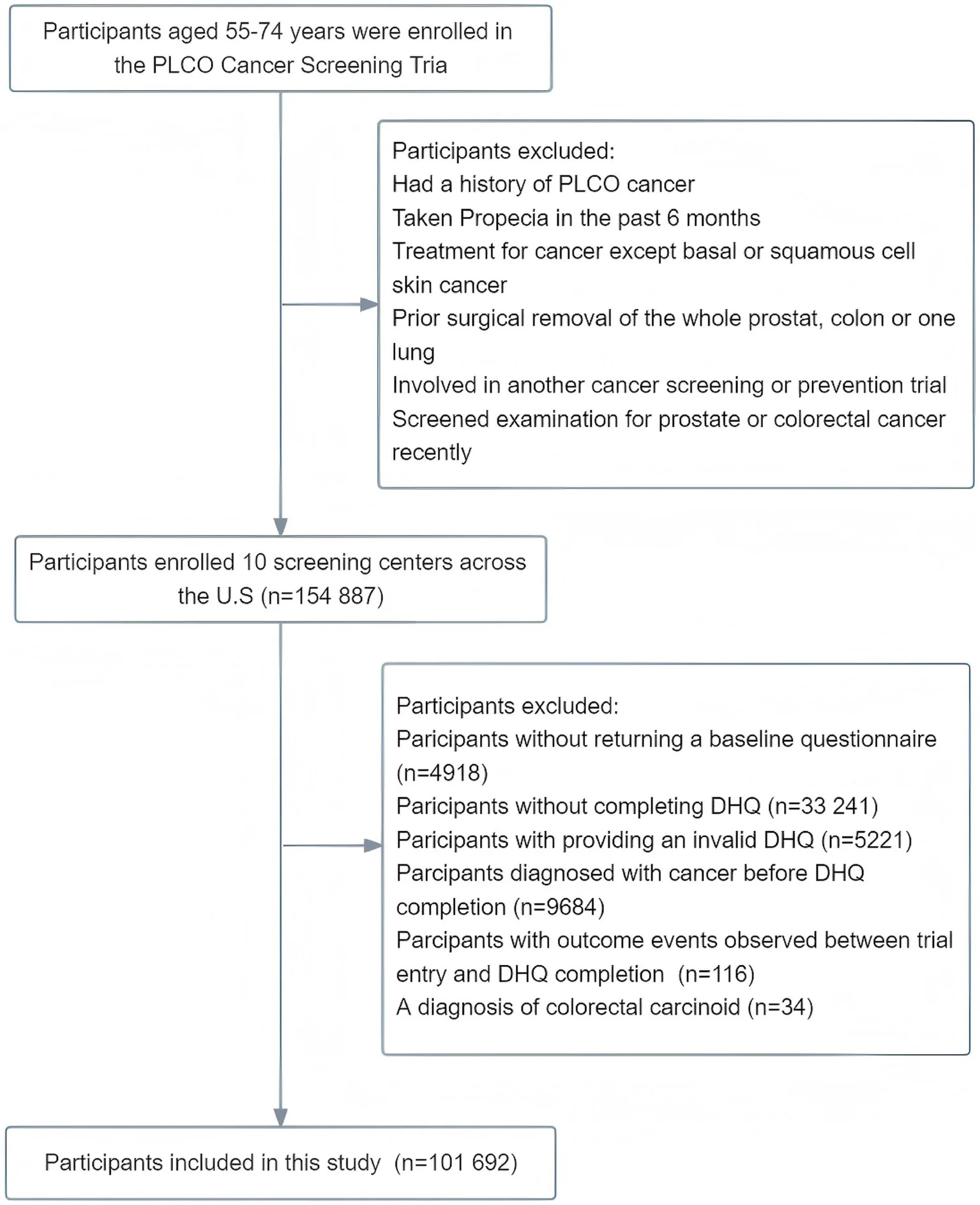
The flow chart of identifying individuals eligible for our study.

Participants were excluded: incomplete baseline questionnaire (*n* = 4,918); missing or invalid dietary data (*n* = 38,462); prior cancer diagnosis before randomization (*n* = 9,684) or an outcome event occurring between randomization and completion of the dietary questionnaire (*n* = 116). The flowchart is presented in Figure 1, and standardized differences between included and excluded participants were all <0.1.

### Outcome ascertainment

In the PLCO Cancer Screening Trial, colorectal cancer (CRC) cases were ascertained through annual study update questionnaires and confirmed by medical record review. Incident CRC was defined using ICD-O-2 codes for the proximal colon (C180-C185), distal colon (C186-C187), and rectum (C199-C209). Cancer diagnoses were collected through 2009. Mortality and cause of death were determined using annual follow-up questionnaires and death certificates, with follow-up through 2018.

### Assessment of covariates and vitamin

**Figure 2:**
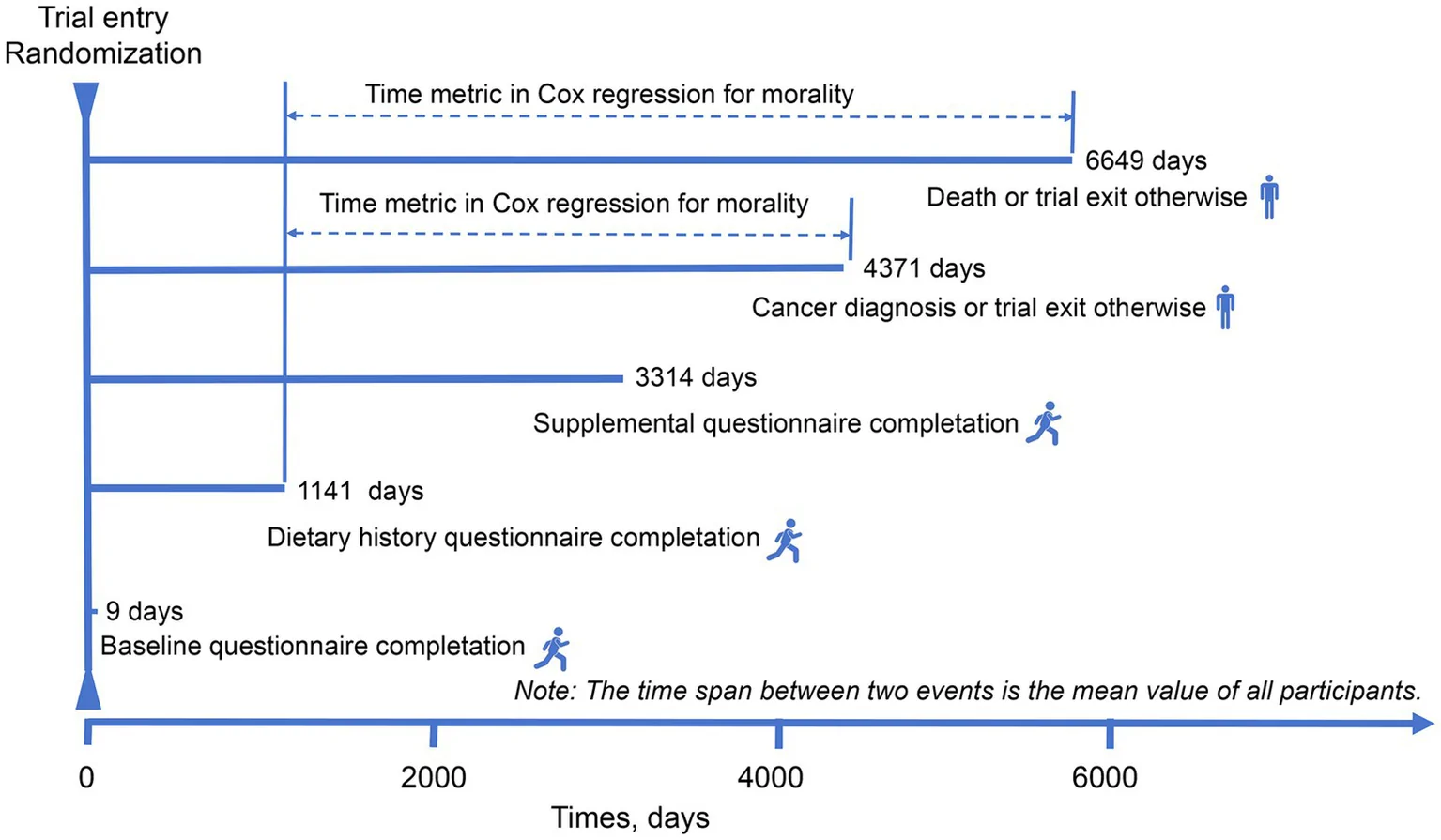
Timeline and follow-up plan for our study. The baseline point for this study was set at the date of completion of the dietary history questionnaire.

In this study, we included age, race, body mass index, marital status, education, physical activity, smoking status, family history of CRC, and history of diabetes and hypertension as covariates. Dietary factors, including total energy intake, alcohol consumption, nutrient intakes (including dietary and supplement sources), and overall diet quality assessed by the Healthy Eating Index-2015, were derived from a validated Diet History Questionnaire in Figure 2. The intake of vitamin were obtained from established national food composition databases. Baseline characteristics are presented in Supplementary Table S2, while missing data/imputation are listed in Supplementary Table S3.

### Statistical analysis

In this study, we conducted Cox proportional hazards regression for the association the intake of various vitamins and CRC incidence and mortality. Model 1 was adjusted for age at DHQ completion, sex education levels and race/ethnicity. Model 2 was adjusted for established variables including lifestyle and clinical factors (BMI, smoking status, pack-years of cigarettes, alcohol consumption, history of colorectal diverticulitis or diverticulosis, colon comorbidities, colorectalpolyp, aspirin use, diabetes, and family history of CRC), physical activity and total energy intake from diet. Model 3 for adjusted for the covariates included in Model 2 plus total protein, carbohydrates, fiber, and sodium. Nonlinearity was tested by examining the null hypothesis that the regression coefficient for the second spline term equaled zero.

Subgroup analyses were conducted stratifying by age (>65 vs. <65 years), BMI (>25 vs. <25), smoking status (current or former vs. never), trial group (screening compared with control groups), and alcohol consumption (≥median vs. <median). Sensitivity analyses were conducted assessing the robustness of the results. All statistical analyses were performed using R software version 4.3.1. Two-sided *p* < 0.05 was considered statistically significant.

## Results

### Baseline characteristics

The study included a total of 101,692 participants, with 49,451 men (48.6%) and 52,241 women (51.4%). The average age of participants was 65.53 (±5.73) years. Baseline characteristics of participants in the PLCO cohort study were classified into specific quartiles based on total vitamin intake (vitamins A, B6, B12, C, D, E) as displayed in Table 1 and Supplementary Table S1. Overall, participants with lower vitamin intake levels tended to less educated, and more likely to have a history of hypertension and aspirin use. They also showed lower levels of physical activity, alcohol consumption, and Healthy Eating Index-2015 scores, but higher body mass index and total energy intake. In terms of dietary composition, these participants consumed higher amounts of fats, carbohydrates, protein, cholesterol, and sodium, along with lower intakes of whole grains, fruits, vegetables, and dietary fiber.

**Table 1:** Baseline characteristics of the PLCO study population according to quarters of vitamin, AB6, B12 (*n* = 101,692), values are mean ± SD or numbers (percentages).

Characteristics	Quarters of vitamin A		*P* for trend^1^	Quarters of vitamin B6		*P* for trend^1^	Quarters of vitamin B12		*P* for trend^1^
	Q1, *N* = 25,424	Q4, *N* = 25,423		Q1, *N* = 25,566	Q4, *N* = 25,406		Q1, *N* = 25,456	Q4, *N* = 25,385	
Age, years	65.3 (5.7)	65.6 (5.7)	<0.001	65.6 (5.8)	65.3 (5.7)	<0.001	65.8 (5.8)	65.4 (5.6)	<0.001
Body mass index (kg/m^2^)	27.5 (4.8)	27.0 (4.9)	<0.001	27.5 (4.8)	27.1 (4.9)	<0.001	27.1 (4.8)	27.6 (4.8)	<0.001
Physical activity (min/week)^2^	104.8 (115.8)	144.7 (131.7)	<0.001	106.6 (115.9)	137.1 (130.2)	<0.001	113.3 (119.7)	131.3 (128.4)	<0.001
Males	14,202 (56.9%)	11,065 (43.5%)		12,616 (49.4%)	12,345 (48.6%)		11,077 (43.5%)	15,119 (59.6%)	
Family history of colorectal cancer, *n* (%)	2,495 (9.8%)	2,525 (9.9%)	<0.001	2,544 (10.0%)	2,539 (10.0%)	<0.001	2,643 (10%)	2,473 (9.7%)	0.002
History of diabetes, *n* (%)	1,817 (7.1%)	1,628 (6.4%)	0.005	1,750 (6.8%)	1,759 (6.9%)	0.005	1,789 (7.0%)	1,861 (7.3%)	<0.001
History of hypertension, *n* (%)	8,606 (33.8%)	7,903 (31.1%)	<0.001	8,535 (33.4%)	8,156 (32.1%)	0.004	8,545 (33.6%)	8,300 (32.7%)	<0.001
Aspirin user, *n* (%)	11,247 (44.2%)	12,281 (48.3%)	<0.001	10,834 (42.4%)	13,078 (51.5%)	<0.001	10,846 (42.6%)	13,195 (52.0%)	<0.001
Body mass index, *n* (%)			<0.001			<0.001			<0.001
Underweight (<18.5 kg/m^2^)	150 (0.6%)	203 (0.8%)		157 (0.6%)	194 (0.8%)		177 (0.7%)	128 (0.5%)	
Normal (18.5–24.9 kg/m^2^)	7,551 (29.7%)	9,170 (36.1%)		7,884 (30.8%)	8,740 (34.4%)		8,750 (34.4%)	7,461 (29.4%)	
Overweight (25–29.9 kg/m^2^)	11,523 (45.3%)	10,566 (41.6%)		11,327 (44.3%)	10,807 (42.5%)		10,956 (43.0%)	11,406 (44.9%)	
Obese (>30 kg/m^2^)	6,200 (24.4%)	5,484 (21.6%)		6,198 (24.2%)	5,665 (22.3%)		5,573 (21.9%)	6,390 (25.2%)	
Racial/ethnic group^3^, *n* (%)			<0.001			<0.001			<0.001
Non-Hispanic White	22,824 (90.0%)	22,876 (90.0%)		22,836 (89.3%)	23,066 (90.8%)		22,251 (87.4%)	23,794 (93.7%)	
Non-Hispanic Black	1,020 (4.0%)	872 (3.4%)		1,135 (4.4%)	754 (3.0%)		1,283 (5.0%)	559 (2.2%)	
Hispanic	467 (1.8%)	350 (1.4%)		425 (1.7%)	429 (1.7%)		449 (1.8%)	327 (1.3%)	
Other race/ethnicity^3^	1,113 (4.4%)	1,325 (5.2%)		1,170 (4.6%)	1,157 (4.6%)		1,473 (5.8%)	705 (2.8%)	
Educational degree, *n* (%)			<0.001			<0.001			<0.001
College below	17,926 (70.5%)	14,736 (58.0%)		17,573 (68.7%)	15,447 (60.8%)		17,242 (67.7%)	15,370 (60.5%)	
College graduate	3,935 (15.5%)	4,746 (18.7%)		4,153 (16.2%)	4,563 (18.0%)		4,125 (16.2%)	4,791 (18.9%)	
Postgraduate	3,563 (14.0%)	5,941 (23.4%)		3,840 (15.0%)	5,396 (21.2%)		4,089 (16.1%)	5,224 (20.6%)	
Smoking status, *n* (%)			<0.001			<0.001			<0.001
Never	11,112 (43.7%)	13,193 (51.9%)		11,891 (46.5%)	12,146 (47.8%)		12,642 (50.0%)	11,356 (44.7%)	
Current	3,265 (12.8%)	1,622 (6.4%)		3,049 (11.9%)	1,974 (7.8%)		2,443 (9.6%)	2,496 (9.8%)	
Former	11,047 (43.5%)	10,608 (41.7%)		10,626 (41.6%)	11,286 (44.4%)		10,371 (40.7%)	11,533 (45.4%)	
Alcohol intake, g/d	10.8 (30.4%)	8.2 (19.2%)	<0.001	7.9 (19.0)	11.8 (34.5)	<0.001	7.8 (21.2)	11.6 (28.2)	<0.001
Energy intake from diet, kcal/day	1,452.2 (620.3)	2,065.7 (800.6)	<0.001	1,392.9 (477.6)	2,065.2 (869.1)	<0.001	1,366.8 (485.1)	2,250.5 (794.4)	<0.001
Healthy eating index-2015	62.3 (9.7)	70.7 (8.5)	<0.001	63.5 (9.8)	69.1 (9.2)	<0.001	65.9 (9.8)	66.6 (9.7)	<0.001
Food consumption									
Whole grain (g/day)	43.7 (46.7)	80.7 (71.9)	<0.001	43.8 (45.9)	77.8 (71.2)	<0.001	52.2 (56.5)	72.0 (63.2)	<0.001
Vegetable (g/day)	173.5 (91.6)	451.7 (237.1)	<0.001	204.4 (111.4)	369.6 (241.8)	<0.001	233.4 (157.8)	347.6 (208.5)	<0.001
Fruit (g/day)	182.4 (152.1)	391.3 (268.6)	<0.001	189.7 (141.2)	360.3 (277.7)	<0.001	241.9 (206.2)	311.7 (231.7)	<0.001
Red Meats (g/day)	56.6 (44.1)	66.0 (61.0)	<0.001	51.2 (35.3)	68.6 (63.5)	<0.001	40.9 (26.1)	92.0 (70.2)	<0.001
White meats (g/day)	35.8 (32.3)	69.8 (61.5)	<0.001	35.7 (30.4)	66.9 (61.6)	<0.001	37.4 (35.6)	70.5 (59.8)	<0.001
Nutrient intake									
Fat (g/day)	53.3 (27.4)	71.6 (38.1)	<0.001	51.6 (23.9)	71.8 (38.7)	<0.001	47.6 (21.6)	83.3 (39.3)	<0.001
Carbohydrate (g/day)	178.6 (72.9)	274.0 (100.5)	<0.001	174.6 (60.4)	267.1 (107.3)	<0.001	181.5 (70.5)	275.9 (97.6)	<0.001
Protein (g/day)	53.0 (22.6)	82.1 (34.6)	<0.001	51.4 (18.1)	80.7 (36.2)	<0.001	48.4 (16.3)	92.0 (33.3)	<0.001
Cholesterol (mg/day)	180.8 (112.2)	235.4 (153.1)	<0.001	174.5 (101.2)	237.4 (155.1)	<0.001	149.0 (79.6)	293.5 (164.1)	<0.001
Dietary fiber (g/day)	12.6 (5.4)	25.4 (9.6)	<0.001	13.0 (4.6)	23.2 (10.4)	<0.001	14.7 (6.9)	22.3 (9.2)	<0.001
Sodium (mg/day)	2,178.5 (895.9)	3,380.1 (1,401.1)	<0.001	2,158.7 (756.9)	3,255.7 (1,441.2)	<0.001	2,096.4 (758.5)	3,611.2 (1,349.3)	<0.001

1 *p*-value for comparison between quarters of total fat intake, by Kruskal-Wallis rank sum test or Pearson’s Chi-squared test.

2 Total time of moderate-to-vigorous physical activity per week.

3 Other race/ethnicity = Asian, Pacific Islander or American Indian.

### Associations between vitamin intake and CRC incidence

During a median follow-up of 8.82 years (896,655 person-years), 1,085 incident colorectal cancer cases were identified, corresponding to an incidence rate of 12.1 per 10,000 person-years. As shown in Table 2, in the fully adjusted model (Model III), higher total intake of several vitamins was associated with lower CRC incidence. Compared with participants in the lowest quartile, those in the highest quartile had lower CRC incidence for vitamin A (HR = 0.75, 95% CI: 0.60–0.93; *P*-trend = 0.005), vitamin B6 (HR = 0.74, 95% CI: 0.61–0.90; *P*-trend = 0.015), vitamin D (HR = 0.77, 95% CI: 0.64–0.93; *P*-trend = 0.022), and vitamin E (HR = 0.81, 95% CI: 0.68–0.97; *P*-trend = 0.008). Vitamin B12 intake was not significantly associated with CRC incidence in the highest quartile compared with the lowest quartile (HR = 0.84, 95% CI: 0.69–1.02; *P*-trend = 0.097). For vitamin C, the highest quartile was also not significantly associated with CRC incidence in the fully adjusted model (HR = 0.89, 95% CI: 0.74–1.07), although the trend test was statistically significant (*P*-trend = 0.007).

**Table 2:** Association all kinds of vitamin by intake source and the incidence of CRC^1^.

Categories	Quartile of proportion of vitamin intake				
	Q1 (*n* = 25,424)	Q2 (*n* = 25,423)	Q3 (*n* = 25,422)	Q4 (*n* = 25,423)	*P-*trend
No of cases	302	293	264	226	
Incidence rate^2^	119	116	104	89	
Total vitamin A					
Model I^3^	1.00 (reference)	0.99 (0.84–1.16)	0.89 (0.75–1.05)	0.77 (0.64–0.91)	0.001
Model II^4^	1.00 (reference)	1.00 (0.85–1.18)	0.90 (0.76–1.08)	0.79 (0.66–0.97)	0.010
Model III^5^	1.00 (reference)	0.99 (0.84–1.17)	0.88 (0.74–1.06)	0.75 (0.60–0.93)	0.005
Person-years	221828.61	223439.68	224699.38	226687.32	
Total vitamin B6					
Model I	1.00 (reference)	0.79 (0.66–0.92)	0.89 (0.74–1.03)	0.75 (0.63–0.89)	0.006
Model II	1.00 (reference)	0.79 (0.66–0.93)	0.89 (0.75–1.05)	0.76 (0.63–0.91)	0.016
Model III	1.00 (reference)	0.78 (0.66–0.93)	0.88 (0.75–1.05)	0.74 (0.61–0.90)	0.015
Person-years	224015.69	223325.50	224576.03	224737.76	
Total vitamin B12					
Model I^3^	1.00 (reference)	0.79 (0.67–0.93)	0.78 (0.66–0.93)	0.84 (0.71–0.97)	0.045
Model II^4^	1.00 (reference)	0.78 (0.66–0.93)	0.80 (0.67–0.94)	0.83 (0.69–1.00)	0.065
Model III^5^	1.00 (reference)	0.79 (0.66–0.93)	0.80 (0.67–0.95)	0.84 (0.69–1.02)	0.097
Person-years	224232.92	224993.27	224385.75	223043.04	
Total vitamin C					
Model I	1.00 (reference)	1.02 (0.87–1.20)	0.82 (0.69–0.98)	0.84 (0.71–1.00)	0.007
Model II	1.00 (reference)	1.06 (0.90–1.24)	0.87 (0.73–1.04)	0.90 (0.75–1.07)	0.071
Model III	1.00 (reference)	1.05 (0.89–1.24)	0.86 (0.71–1.04)	0.89 (0.74–1.07)	0.007
Person-years	220594.15	223545.20	225454.07	227061.56	
Total vitamin D					
Model I	1.00 (reference)	0.76 (0.64–0.89)	0.82 (0.70–0.97)	0.76 (0.65–0.90)	0.007
Model II	1.00 (reference)	0.75 (0.63–0.89)	0.84 (0.71–0.99)	0.77 (0.65–0.92)	0.017
Model III	1.00 (reference)	0.75 (0.63–0.89)	0.84 (0.71–0.99)	0.77 (0.64–0.93)	0.022
Person-years	223629.55	224924.19	224721.77	223379.47	
Total vitamin E					
Model I	1.00 (reference)	0.91 (0.78–1.07)	0.78 (0.66–0.93)	0.76 (0.64–0.90)	0.001
Model II	1.00 (reference)	0.93 (0.79–1.10)	0.82 (0.69–0.97)	0.80 (0.68–0.96)	0.005
Model III	1.00 (reference)	0.94 (0.80–1.11)	0.83 (0.70–0.98)	0.81 (0.68–0.97)	0.008
Person-years	221634.27	221769.71	226717.13	226533.88	

1 Values are hazard ratios (95% confidence intervals).

2 Crude incidence rate per 10 000 person-years.

3 Age (continuous), sex (male, female), race (non-Hispanic White, Non-Hispanic Black, Hispanic, other race/ethnicity), education levels (some college or less, college graduate, postgraduate).

4 Family history of colorectal cancer (no, yes or possibly), history of colon comorbidity (no, yes), history of diverticulitis or diverticulosis (no, yes), history of colorectal polyp (no, yes), history of diabetes (no, yes), history of aspirin use (no, yes), total energy intake (continuous), body mass index at baseline (continuous), smoking status (never, current, former), alcohol consumption (continuous), and physical activity level (continuous), cigarette.

5 Adjusted for covariates in model 2 plus carbohydrates, protein, dietary fiber, sodium.

**Figure 3:**
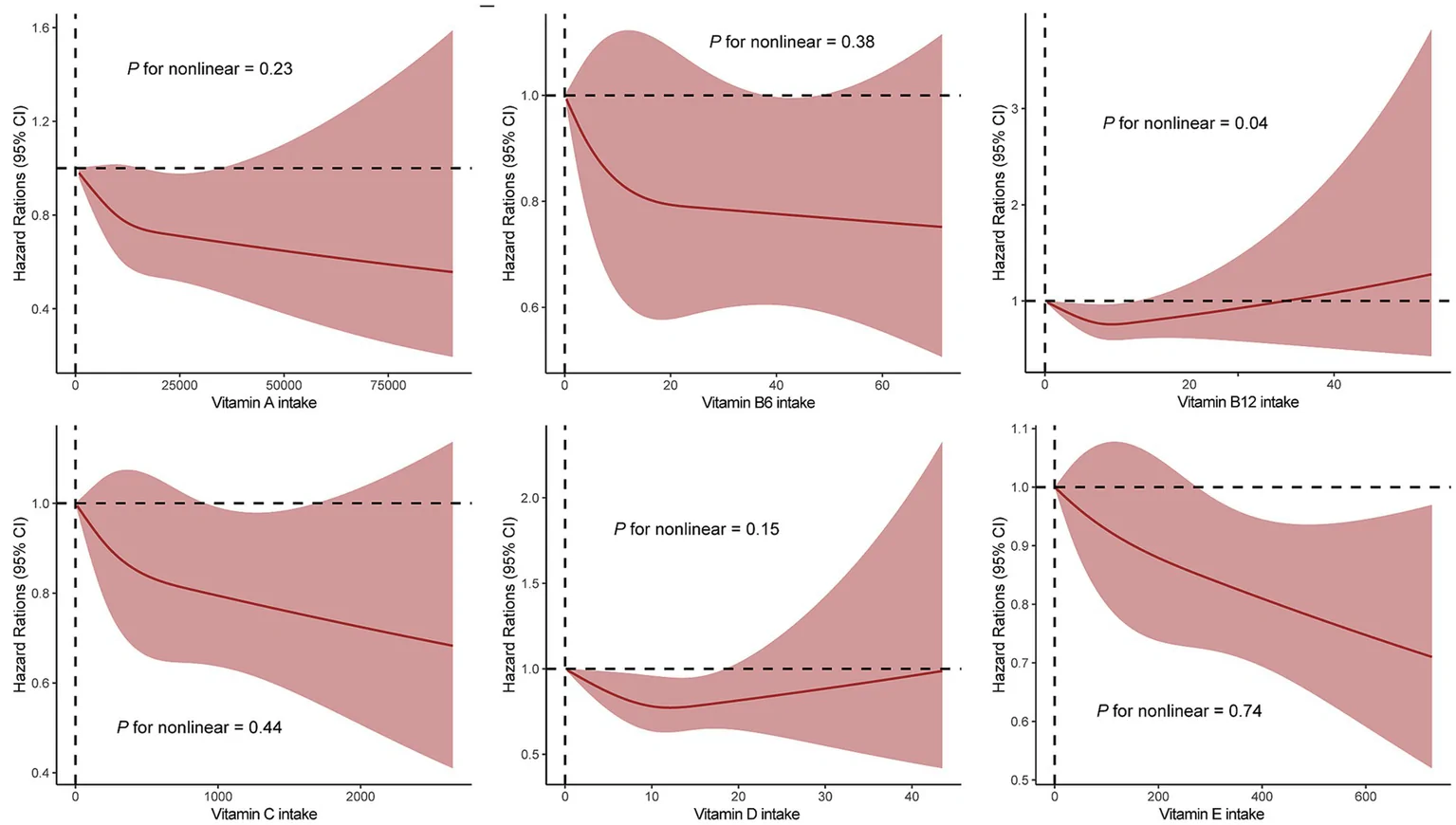
Restricted cubic spline analyses to assess potential nonlinear associations between vitamin intake and colorectal cancer incidence.

Restricted cubic spline analyses were used to explore potential nonlinear dose–response relationships. Evidence of nonlinearity was observed only for vitamin B12 (P for nonlinearity = 0.04). No clear evidence of nonlinearity was observed for vitamin A, B6, C, D, or E in Figure 3.

Subgroup analyses indicated possible heterogeneity across certain population characteristics. For example, A stronger inverse association for vitamin A was observed in men (HR = 0.58, 95% CI: 0.42–0.80; *P*-trend = 0.012), whereas vitamin C showed a stronger association in women (HR = 0.72, 95% CI: 0.54–0.94; *P*-trend = 0.018). In addition, vitamin A (HR = 0.62, 95% CI: 0.46–0.84; P-trend = 0.004) and vitamin D (HR = 0.66, 95% CI: 0.50–0.86; *P*-trend = 0.021) showed stronger associations among non-aspirin users compared with aspirin users (Supplementary Tables S4–S5). Sensitivity analyses yielded generally consistent results (Supplementary Tables S8–S9).

### Associations between vitamin intake and CRC mortality

During a median follow-up of 15.08 years (1,533,506 person-years), 529 CRC-related deaths were recorded, with an overall mortality rate of 3.5 per 10,000 person-years (Table 3). In the fully adjusted model (Model III), higher intake of several vitamins was associated with lower CRC mortality. Specifically, participants in the highest quartile (Q4) had lower mortality compared with those in the lowest quartile (Q1) for vitamin A (HR = 0.74, 95% CI: 0.55–1.00; *P*-trend = 0.016), vitamin B6 (HR = 0.73, 95% CI: 0.55–0.97; *P*-trend = 0.048), vitamin C (HR = 0.77, 95% CI: 0.59–1.00; *P*-trend = 0.047), vitamin D (HR = 0.57, 95% CI: 0.43–0.75; *P-*trend = 0.001), and vitamin E (HR = 0.73, 95% CI: 0.57–0.95; *P*-trend = 0.009). In contrast, vitamin B12 intake was not significantly associated with CRC mortality in the fully adjusted model (HR = 0.81, 95% CI: 0.61–1.08; *P*-trend = 0.078).

**Table 3:** Association all kinds of vitamin by intake source and the mortality of CRC^1^.

	Quartile of proportion of vitamin intake				
	Q1 (*n* = 25,424)	Q2 (*n* = 25,423)	Q3 (*n* = 25,422)	Q4 (*n* = 25,423)	*P-*trend
No of cases	158	147	110	114	
Incidence rate^2^	63	58	44	45	
Total vitamin A					
Model I^3^	1.00 (reference)	0.93 (0.74–1.12)	0.68 (0.53–0.87)	0.70 (0.55–0.89)	0.001
Model II^4^	1.00 (reference)	0.95 (0.76–1.20)	0.72 (0.55–0.92)	0.75 (0.56–0.98)	0.009
Model III^5^	1.00 (reference)	0.96 (0.76–1.21)	0.72 (0.55–0.93)	0.74 (0.55–1.00)	0.016
Person-years	375954.46	380698.85	385237.39	391615.57	
Total vitamin B6					
Model I	1.00 (reference)	0.81(0.64–1.02)	0.82(0.65–1.03)	0.70(0.55–0.89)	0.007
Model II	1.00 (reference)	0.83 (0.65–1.05)	0.85 (0.67–1.08)	0.72 (0.55–0.94)	0.025
Model III	1.00 (reference)	0.83 (0.65–1.06)	0.86 (0.68–1.10)	0.73 (0.55–0.97)	0.048
Person-years	380441.24	381922.73	385136.55	386005.76	
Total vitamin B12					
Model I	1.00 (reference)	0.90 (0.72–1.13)	0.74 (0.58–0.95)	0.79 (0.62–1.00)	0.020
Model II	1.00 (reference)	0.90 (0.71–1.14)	0.76 (0.59–0.98)	0.79 (0.60–1.03)	0.037
Model III	1.00 (reference)	0.91 (0.72–1.16)	0.77 (0.60–0.99)	0.81 (0.61–1.08)	0.078
Person-years	382361.66	384929.00	385347.03	380868.58	
Total vitamin C					
Model I	1.00 (reference)	0.83 (0.66–1.04)	0.74 (0.58–0.94)	0.67 (0.53–0.86)	0.001
Model II	1.00 (reference)	0.88(0.70–1.11)	0.82(0.64–1.05)	0.75(0.58–0.96)	0.020
Model III	1.00 (reference)	0.89(0.70–1.13)	0.84(0.64–1.09)	0.77(0.59–1.00)	0.047
Person-years	375586.70	382878.91	387540.28	387500.39	
Total vitamin D					
Model I	1.00 (reference)	0.71 (0.56–0.90)	0.84 (0.67–1.06)	0.56 (0.43–0.72)	0.000
Model II	1.00 (reference)	0.70 (0.55–0.89)	0.87 (0.69–1.09)	0.56 (0.43–0.74)	0.000
Model III	1.00 (reference)	0.70 (0.55–0.90)	0.87 (0.69–1.09)	0.57 (0.43–0.75)	0.001
Person-years	381716.52	384100.45	386057.78	381631.52	
Total vitamin E					
Model I	1.00 (reference)	0.87 (0.69–1.09)	0.72 (0.57–0.91)	0.66 (0.51–0.84)	0.000
Model II	1.00 (reference)	0.91 (0.72–1.14)	0.78 (0.61–0.99)	0.71 (0.55–0.92)	0.004
Model III	1.00 (reference)	0.92 (0.73–1.15)	0.69(0.62–1.01)	0.73 (0.57–0.95)	0.009
Person-years	376413.19	380374.14	388882.12	387836.84	

1 Values are hazard ratios (95% confidence intervals).

2 Crude incidence rate per 10,000 person-years.

3 Age (continuous), sex (male, female), race (non-Hispanic white, non-Hispanic black, Hispanic, other race/ethnicity), education levels (some college or less, college graduate, postgraduate).

4 Family history of colorectal cancer (no, yes or possibly), history of colon comorbidity (no, yes), history of diverticulitis or diverticulosis (no, yes), history of colorectal polyp (no, yes), history of diabetes (no, yes), history of aspirin use (no, yes), total energy intake (continuous), body mass index at baseline (continuous), smoking status (never, current, former), alcohol consumption (continuous), and physical activity level (continuous), cigarette.

5 Adjusted for covariates in model 2 plus carbohydrates, protein, dietary fiber, sodium.

**Figure 4:**
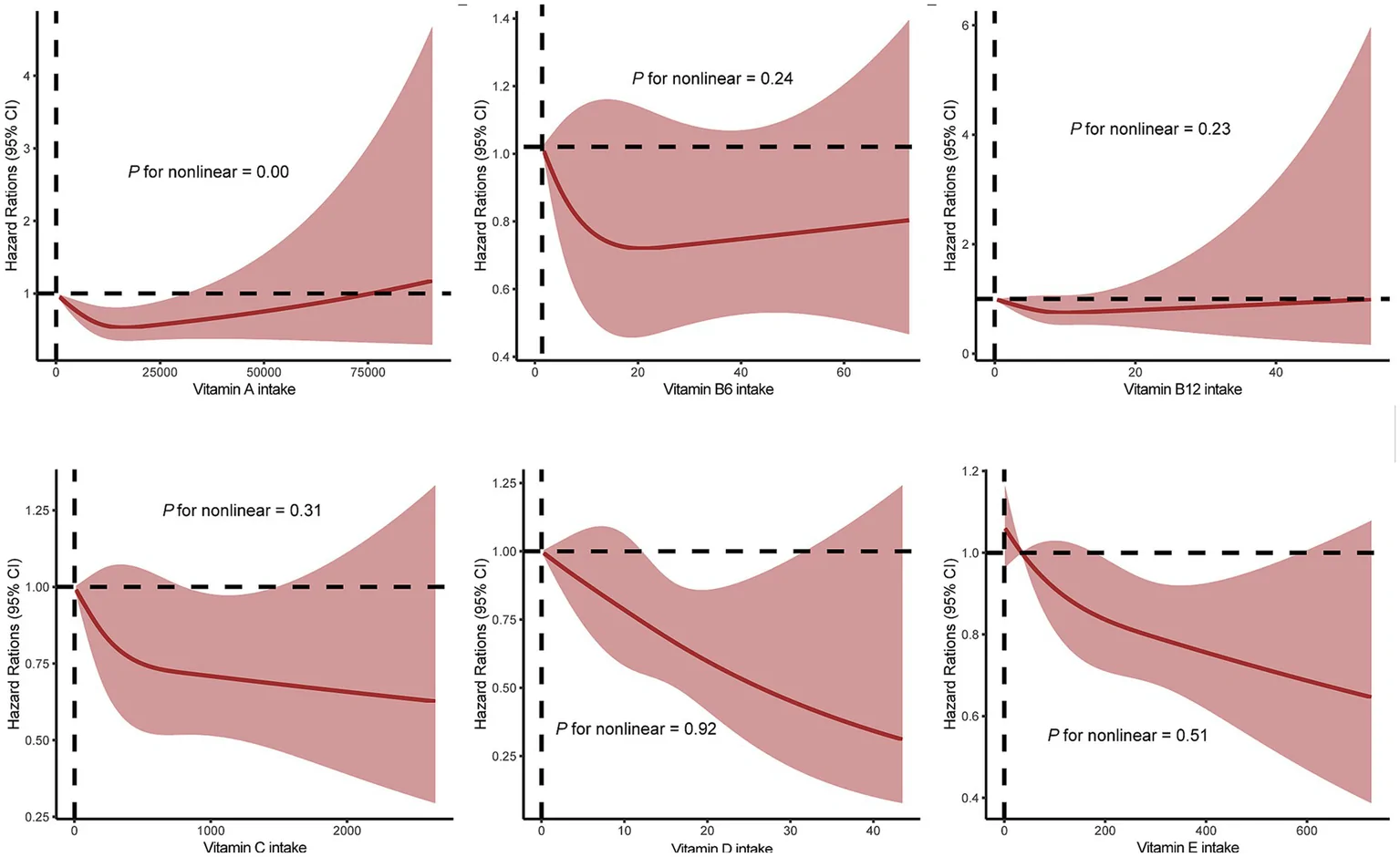
Restricted cubic spline analyses to assess potential nonlinear associations between vitamin intake and mortality of CRC.

Figure 4 were performed to explore potential nonlinear dose–response relationships between vitamin intake and CRC mortality. A statistically significant nonlinear association was observed for vitamin A (P for nonlinearity <0.001), suggesting a curved relationship across intake levels. For the other vitamins, including vitamins B6, B12, C, D, and E, there was no strong evidence of nonlinearity, and the associations appeared broadly consistent with linear trends. Subgroup analyses indicated possible effect modification by sex, aspirin use, and smoking status (Supplementary Tables S6–S7). Sensitivity analyses produced generally consistent results across alternative model specifications (Supplementary Tables S10–S11).

## Discussion

In this large prospective multicentre cohort study, higher intakes of vitamins A, B6, C, D, and E were observed to be inversely associated with CRC incidence and mortality, with evidence of dose–response patterns for most vitamins. In contrast, no clear associations were observed for vitamin B12 intake with either CRC incidence or mortality. These associations showed general consistency across multiple analytic frameworks, including subgroup analyses and a series of sensitivity analyses accounting for early events, extreme body mass index values, and alternative dietary adjustments. Overall, these results suggest that total vitamin intake from both dietary and supplemental sources may be correlated with CRC incidence and mortality in this cohort.

### Interpretation and comparison with other studies

Based on NHANES data, approximately 31% of adults use multivitamin supplements, highlighting the widespread exposure to vitamin supplementation (13). Previous epidemiological studies have largely focused on single-nutrient analyses, in contrast, the present analysis simultaneously evaluated multiple vitamins including A, B6, B12, C, D, and E within a single well-characterized prospective cohort, enabling a more systematic comparison of their associations under identical methodological conditions.

Vitamin A deficiency can lead to hirschsprung disease (14). Increasing evidence suggests that vitamin C acts as an anticancer agent (15), such as, high-dose vitamin C in combination with chemotherapy potentially benefiting patients with metastatic colorectal cancer (mCRC) carrying RAS (KRAS, NRAS, and HRAS) mutations (16). Vitamin E is associated with an increased risk of recurrence in non-muscle invasive bladder cancer (NMIBC) (17). Selenium or vitamin E supplementation might offer protection against prostate cancer risk (18), Vitamin E Supplementation in patients enhances cancer immunotherapy through dendritic cell function (19). Additionally, low serum vitamin B6 levels are associated with a higher risk of cardiovascular disease and all-cause mortality in the elderly population (20). Higher total vitamin D intake was associated with a lower risk of early-onset CRC (7). Our findings are broadly consistent with prior observational studies reporting inverse associations for selected antioxidant vitamins (21), although randomized controlled trials of vitamin supplementation have not consistently demonstrated protective effects on cancer outcomes. For instance, the D-Health Trial, did not reported that vitamin D supplementation was not associated with a reduced risk of total cancer incidence or site-specific cancers, including colorectal cancer (22). Long-term follow-up data from randomized trials of calcium and vitamin D supplementation in postmenopausal women have reduced in cancer mortality, and suggest possible divergent effects on cancer and cardiovascular outcomes (23). Furthermore, we will acknowledge that previous large-scale supplementation trials, such as the SELECT trial, which failed to confirm observational associations for vitamin E and instead reported an increased risk of prostate cancer (24). These discrepancies may reflect differences between dietary intake patterns and pharmacologic supplementation, as well as the influence of underlying dietary and lifestyle confounding.

Unlike other vitamins examined, vitamin B12 intake was not significantly associated with colorectal cancer incidence or mortality in this cohort. However, one study has suggested that folic acid and vitamin B12 supplementation may increase the risk of developing colorectal cancer (25). Although a non-significant trend was observed in some analyses, the lack of statistical robustness does not support definitive interpretation.

Prior studies indicate that estrogen and free testosterone reduces CRC risk (26, 27). Our research revealed that the protective link between sex and lower CRC incidence was mainly observed in men taking vitamin A (Supplementary Table S4: HR_quartile_ 4 vs. 1: 0.58; 95% CI: 0.42–0.80; *P*_interaction_ = 0.012) and in women taking vitamin C (Supplementary Table S5: HR_quartile_ 4 vs. 1: 0.72; 95% CI: 0.54–0.94; *P*_interaction_ = 0.018). This finding indicates that vitamins A and C intake could potentially diminish CRC incidence by modulating sex hormone levels.

From a public health perspective, our results suggest that total vitamin intake, as part of broader dietary patterns, may be associated with colorectal cancer risk and mortality. They reinforce the importance of balanced dietary patterns rich in plant-based foods and micronutrient diversity, consistent with established dietary guidelines.

### Strengths and limitations

This study has several notable strengths. Leveraging data from the PLCO trial, we utilized a large prospective cohort with long-term follow-up, high-quality ascertainment of CRC incidence and mortality, and detailed assessments of vitamin intake and relevant covariates. However, certain limitations must also be acknowledged. Although we rigorously adjusted for the identified risk factors, potential residual and unmeasured confounding cannot be completely ruled out due to the observational study design we adopted. Additionally, this observational design prevents us from establishing causal relationships between the examined factors and outcomes. Additionally, the use of repeated and comprehensive covariate adjustment and multiple sensitivity analyses enhances the robustness of our findings.

However, several limitations should be considered. First, the population consisted predominantly of well-nourished and generally healthy individuals, which may limit the applicability of our findings to populations with nutritional deficiencies. Second, despite extensive adjustment for potential confounders, residual confounding cannot be entirely excluded due to the observational nature of the study. Third, we focused primarily on individual vitamins rather than specific multiple vitamins formulations or combined supplementation patterns; concurrent use of multiple supplements may have influenced the observed associations. Finally, we did not evaluate biomarkers of vitamin status, which may more accurately reflect biological exposure than dietary intake alone.

## Conclusion

In general, higher dietary intake of most vitamins was found to be negative associated with CRC incidence and mortality. Due to the observational nature of the study, no causal inference can be established. Nevertheless, these findings highlight the relevance of considering vitamin consumption as part of comprehensive dietary evaluation frameworks. Further evidence from well-designed epidemiological studies and experimental research is needed to verify these associations, particularly in large and diverse populations. If these observations are confirmed, they may contribute to a better understanding of the potential role of overall vitamin exposure in CRC-related public health strategies.

## Data Availability

The original contributions presented in the study are included in the article/Supplementary material, further inquiries can be directed to the corresponding authors.

## References

[1] Sung H Ferlay J Siegel RL Laversanne M Soerjomataram I Jemal A . Global cancer statistics 2020: GLOBOCAN estimates of incidence and mortality worldwide for 36 cancers in 185 countries. CA Cancer J Clin. (2020) 71:209–49. doi: 10.3322/caac.21660, 33538338

[2] Bray F Laversanne M Sung H Ferlay J Siegel RL Soerjomataram I . Global cancer statistics 2022: GLOBOCAN estimates of incidence and mortality worldwide for 36 cancers in 185 countries. CA Cancer J Clin. (2022) 74:229–63. doi: 10.3322/caac.21834, 38572751

[3] Sawicki T Ruszkowska M Danielewicz A Niedźwiedzka E Arłukowicz T Przybyłowicz KE. A review of colorectal cancer in terms of epidemiology, risk factors, development, symptoms and diagnosis. Cancers (Basel). (2021) 13:2025. doi: 10.3390/cancers13092025, 33922197 PMC8122718

[4] Papadimitriou N Markozannes G Kanellopoulou A Critselis E Alhardan S Karafousia V . An umbrella review of the evidence associating diet and cancer risk at 11 anatomical sites. Nat Commun. (2021) 12:4579. doi: 10.1038/s41467-021-24861-8 PMC8319326 34321471

[5] Song M Chan AT Sun J. Influence of the gut microbiome, diet, and environment on risk of colorectal cancer. Gastroenterology. (2020) 158:322–40. doi: 10.1053/j.gastro.2019.06.048, 31586566 PMC6957737

[6] DJA J Spence JD Giovannucci EL Kim YI Josse RG Vieth R . Supplemental vitamins and minerals for cardiovascular disease prevention and treatment: JACC focus seminar. J Am Coll Cardiol. (2021) 77:423–36. doi: 10.1016/j.jacc.2020.09.619 33509399

[7] Kim H Lipsyc-Sharf M Zong X Wang X Hur J Song M . Total vitamin D intake and risks of early-onset colorectal cancer and precursors. Gastroenterology. (2021) 161:1208–1217.e9. doi: 10.1053/j.gastro.2021.07.002, 34245763 PMC8463427

[8] Shi S Wang K Ugai T Giannakis M Cazaubiel J Chan AT . Vitamin C intake and colorectal cancer survival according to KRAS and BRAF mutation: a prospective study in two US cohorts. Br J Cancer. (2023) 129:1793–800. doi: 10.1038/s41416-023-02452-2, 37775523 PMC10667518

[9] Bellerba F Serrano D Harriet J Pozzi C Segata N NabiNejad A . Colorectal cancer, vitamin D and microbiota: a double-blind phase II randomized trial (ColoViD) in colorectal cancer patients. Neoplasia. (2022) 34:100842. doi: 10.1016/j.neo.2022.100842 PMC9594107 36279751

[10] Li J Qin S Zhang S Lu Y Shen Q Cheng L . Serum vitamin D concentration, vitamin D-related polymorphisms, and colorectal cancer risk. Int J Cancer. (2023) 153:278–89. doi: 10.1002/ijc.34521, 36946647

[11] Andersen V Halekoh U Bohn T Tjønneland A Vogel U Kopp TI. No interaction between polymorphisms related to vitamin a metabolism and vitamin A intake in relation to colorectal cancer in a prospective Danish cohort. Nutrients. (2019) 11. doi: 10.3390/nu11061428, 31242605 PMC6627526

[12] Sesso HD Manson JE Aragaki AK Rist PM Johnson LG Friedenberg G . Effect of cocoa flavanol supplementation for the prevention of cardiovascular disease events: the COcoa supplement and multivitamin outcomes study (COSMOS) randomized clinical trial. Am J Clin Nutr. (2022) 115:1490–500. doi: 10.1093/ajcn/nqac055, 35294962 PMC9170467

[13] Zhang Y Fang F Tang J Jia L Feng Y Xu P . Association between vitamin D supplementation and mortality: systematic review and meta-analysis. BMJ. (2019) 366:l4673. doi: 10.1136/bmj.l4673, 31405892 PMC6689821

[14] Hegde SG Devi S Sivadas A Shubha AM Thomas A Mukhopadhyay A . Maternal vitamin a status as a risk factor of Hirschsprung disease in the child. Clin Transl Gastroenterol. (2023) 14:e00619. doi: 10.14309/ctg.0000000000000619, 37490568 PMC10522106

[15] Magrì A Germano G Lorenzato A Lamba S Chilà R Montone M . High-dose vitamin C enhances cancer immunotherapy. Sci Transl Med. (2020) 12. doi: 10.1126/scitranslmed.aay8707 32102933

[16] Wang F He MM Xiao J Zhang Y-Q Yuan X-L Fang W-J . A randomized, open-label, multicenter, phase 3 study of high-dose vitamin C plus FOLFOX ± bevacizumab versus FOLFOX ± bevacizumab in unresectable untreated metastatic colorectal cancer (VITALITY study). Clin Cancer Res. (2022) 28:4232–9. doi: 10.1158/1078-0432.ccr-22-0655, 35929990 PMC9527503

[17] Bryan RT Pirrie SJ Abbotts B Maycock S During V Lewis C . Selenium and vitamin E for prevention of non-muscle-invasive bladder cancer recurrence and progression: a randomized clinical trial. JAMA Netw Open. (2023) 6:e2337494. doi: 10.1001/jamanetworkopen.2023.37494 PMC10582794 37847504

[18] Loh WQ Youn J Seow WJ. Vitamin E intake and risk of prostate cancer: a meta-analysis. Nutrients. (2022) 15. doi: 10.3390/nu15010014, 36615673 PMC9824720

[19] Yuan X Duan Y Xiao Y Sun K Qi Y Zhang Y . Vitamin E enhances cancer immunotherapy by reinvigorating dendritic cells via targeting checkpoint SHP1. Cancer Discov. (2022) 12:1742–59. doi: 10.1158/2159-8290.CD-21-0900, 35420681 PMC9262841

[20] Wang P Huang J Xue F Abuduaini M Tao Y Liu H. Associations of serum vitamin B6 status with the risks of cardiovascular, cancer, and all-cause mortality in the elderly. Front Immunol. (2024) 15:1354958. doi: 10.3389/fimmu.2024.1354958, 38698865 PMC11064647

[21] Mangione CM Barry MJ Nicholson WK US Preventive Services Task Force Cabana M Chelmow D . Vitamin, mineral, and multivitamin supplementation to prevent cardiovascular disease and cancer: US preventive services task force recommendation statement. JAMA. (2022) 327:2326–33. doi: 10.1001/jama.2022.8970, 35727271

[22] Neale RE English DR Mcleod DS Armstrong BK Baxter C Romero BD . The effect of vitamin D supplementation on cancer incidence in the randomised controlled D-health trial: implications for policy and practice. J Steroid Biochem Mol Biol. (2025) 250:106738. doi: 10.1016/j.jsbmb.2025.106738, 40096917

[23] Thomson CA Aragaki AK Prentice RL Stefanick ML Manson JE Wactawski-Wende J . Long-term effect of randomization to calcium and vitamin D supplementation on health in older women: postintervention follow-up of a randomized clinical trial. Ann Intern Med. (2024) 177:428–38. doi: 10.7326/M23-2598 38467003

[24] Klein EA Thompson IM Tangen CM Crowley JJ Lucia MS Goodman PJ . Vitamin E and the risk of prostate cancer: the selenium and vitamin E cancer prevention trial (SELECT). JAMA. (2011) 306:1549–56. doi: 10.1001/jama.2011.1437 PMC4169010 21990298

[25] Oliai Araghi S Kiefte-de Jong JC van Dijk SC KMA S van Laarhoven HW van Schoor NM . Folic acid and vitamin B12 supplementation and the risk of cancer: long-term follow-up of the B vitamins for the prevention of osteoporotic fractures (B-PROOF) trial. Cancer Epidemiol Biomarkers Prev. (2019) 28:275–82. doi: 10.1158/1055-9965.EPI-17-1198, 30341095

[26] Wu Z Huang Y Zhang R Zheng C You F Wang M . Sex differences in colorectal cancer: with a focus on sex hormone-gut microbiome axis. Cell Commun Signal. (2024) 22:167. doi: 10.1186/s12964-024-01549-2, 38454453 PMC10921775

[27] Yang W Giovannucci EL Hankinson SE Chan AT Ma Y Wu K . Endogenous sex hormones and colorectal cancer survival among men and women. Int J Cancer. (2020) 147:920–30. doi: 10.1002/ijc.32844, 31863463 PMC7895324

