# Supplementary material for: Vitamin intake and colorectal cancer incidence and mortality: evidence from a large prospective cohort
Source: Front Nutr. 2026 Sep 9;13:1877860. doi: 10.3389/fnut.2026.1877860 (PMC13597460; doi:10.3389/fnut.2026.1877860)
Supplement: Supplementary file 1 [file Table_1.docx]

**Supplementary Table S1.** Baseline characteristics of the PLCO study population according to sex specific quarters of vitamin C、D、E (n = 101,692),

*Values are mean ± SD or numbers (percentages)

| **Characteristics** | **Quarters of vitamin C** | | ***P* for trend**^1^ | **Quarters of vitamin D** | | ***P* for trend^1^** | **Quarters of vitamin E** | | | ***P* for trend^1^** |
| --- | --- | --- | --- | --- | --- | --- | --- | --- | --- | --- |
|  | **Q1**, N = 25,430 | **Q4**, N = 25,423 |  | **Q1**, N = 25,449 | **Q4**, N = 25,364 |  | **Q1**, N = 25,428 | | **Q4**, N = 25,412 |  |
| Age, years | 65.2 (5.7) | 65.7 (5.7) | <0.001 | 65.5 (5.8) | 65.7 (5.7) | <0.001 | 65.4 (5.8) | | 65.7 (5.6) | <0.001 |
| Body mass index (kg/m^2^) | 27.6 (4.7) | 26.8 (4.8) | <0.001 | 27.3 (4.8) | 27.2 (4.7) | <0.001 | 27.8 (4.9) | | 26.9 (4.7) | <0.001 |
| Physical activity (min/week)^2^ | 103.7 (115.1) | 134.1 (128.3) | <0.001 | 114.0 (120.7) | 132.5 (128.0) | <0.001 | 112.1 (119.9) | | 132.9 (127.1) | <0.001 |
| Males | 14,223 (55.9%) | 11,144 (43.8%) |  | 12,294 (48.3%) | 13,170 (51.9%) |  | 14,754 (58.0%) | | 10,758 (42.3%) |  |
| Family history of Colorectal Cancer, n (%) | 2,497 (9.8%) | 2,526 (9.9%) | <0.001 | 2,605 (10%) | 2,522 (9.9%) | 0.092 | 2,509 (9.9%) | | 2,566 (10%) | <0.001 |
| History of diabetes, n (%) | 1,889 (7.4%) | 1,549 (6.1%) | <0.001 | 1,821 (7.2%) | 1,703 (6.7%) | <0.001 | 1,962 (7.7%) | | 1,538 (6.1%) | <0.001 |
| History of hypertension, n (%) | 8,279 (32.6%) | 8,158 (32.1%) | 0.11 | 8,702 (34.2%) | 8,154 (32.1%) | <0.001 | 8,550 (33.6%) | | 8,423 (33.1%) | <0.001 |
| Aspirin user, n (%) | 10,776 (42.4%) | 13,254 (52.1%) | <0.001 | 10,964 (43.1%) | 13,113 (51.7%) | <0.001 | 10,252 (40.3%) | | 13,528 (53.2%) | <0.001 |
| **Body mass index, n (%)** |  |  | <0.001 |  |  | <0.001 |  | |  | <0.001 |
| Underweight (<18.5 kg/m^2^) | 149 (0.6%) | 187 (0.7%) |  | 166 (0.7%) | 145 (0.6%) |  | 139 (0.5%) | | 172 (0.7%) |  |
| Normal (18.5-24.9 kg/m^2^) | 7,364 (29.0%) | 9,482 (37.3%) |  | 8,234 (32.4%) | 8,403 (33.1%) |  | 7,184 (28.3%) | | 9,412 (37.0%) |  |
| Overweight (25-29.9 kg/m^2^) | 11,634 (45.8%) | 10,615 (41.8%) |  | 11,066 (43.5%) | 11,069 (43.6%) |  | 11,429 (45.0%) | | 10,594 (41.7%) |  |
| Obese (>30 kg/m^2^) | 6,283 (24.7%) | 5,139 (20.2%) |  | 5,983 (23.5%) | 5,747 (23.5%) |  | 6,676 (26.3%) | | 5,234 (20.6%) |  |
| **Racial/ethnic group^3^, n (%)** |  |  | <0.001 |  |  | <0.001 |  | |  | <0.001 |
| Non-Hispanic White | 22,962 (90.3%) | 23,106 (90.9%) |  | 22,265 (87.5%) | 23,840 (94.0%) |  | 22,788 (89.6%) | | 23,533 (92.6%) |  |
| Non-Hispanic Black | 913 (3.6%) | 609 (2.4%) |  | 1,281 (5.0%) | 491 (1.9%) |  | 1,201 (4.7%) | | 460 (1.8%) |  |
| Hispanic | 395 (1.6%) | 352 (1.4%) |  | 480 (1.9%) | 280 (1.1%) |  | 445 (1.8%) | | 310 (1.2%) |  |
| Other race/ethnicity ^3^ | 1,160 (4.6%) | 1,356 (5.3%) |  | 1,423 (5.6%) | 753 (3.0%) |  | 994 (3.9%) | | 1,109 (4.4%) |  |
| **Educational degree, n (%)** |  |  | <0.001 |  |  | <0.001 |  | |  | <0.001 |
| College below | 18,021 (70.9%) | 15,303 (60.2%) |  | 17,392 (68.3%) | 15,261 (60.2%) |  | 17,301 (68.0%) | | 15,365 (60.5%) |  |
| College graduate | 3,922 (15.4%) | 4,749 (18.7%) |  | 4,013 (15.8%) | 4,818 (19.0%) |  | 4,144 (16.3%) | | 4,753 (18.7%) |  |
| Postgraduate | 3,487 (13.7%) | 5,371 (21.1%) |  | 4,044 (15.9%) | 5,285 (20.8%) |  | 3,983 (15.7%) | | 5,294 (20.8%) |  |
| **Smoking status, n (%)** |  |  | <0.001 |  |  | <0.001 |  | |  | <0.001 |
| Never | 10,761 (42.3%) | 12,094 (47.6%) |  | 11,908 (46.8%) | 12,228 (48.2%) |  | 11,609 (45.7%) | | 12,077 (47.5%) |  |
| Current | 3,546 (13.9%) | 1,934 (7.6%) |  | 2,630 (10.3%) | 2,156 (8.5%) |  | 3,053 (12.0%) | | 1,773 (7.0%) |  |
| Former | 11,123 (43.7%) | 11,395 (44.8%) |  | 10,911 (42.9%) | 10,980 (43.3%) |  | 10,766 (42.3%) | | 11,562 (45.5%) |  |
| Alcohol intake, g/d | 10.8 (30.3) | 9.0 (22.2 | <0.001 | 9.8 (27.0) | 9.3 (22.8) | <0.001 | 10.5 (28.3) | | 9.1 (22.6) | <0.001 |
| Energy intake from diet, kcal/day | 1,520.3 (677.7) | 1,780.4 (737.0) | <0.001 | 1,467.4 (584.0) | 2,058.6 (757.6) | <0.001 | 1,614.7 (627.9) | | 1,763.6 (692.5) | <0.001 |
| Healthy Eating Index-2015 | 61.3 (9.8) | 69.0 (9.2) | <0.001 | 64.6 (9.7) | 68.5 (9.6) | <0.001 | 63.6 (9.7) | | 69.0 (9.2) | <0.001 |
| **Food consumption** |  |  |  |  |  |  |  | |  |  |
| Whole grain (g/day) | 45.1 (48.4) | 68.4 (65.8) | <0.001 | 50.7 (56.6) | 72.5 (62.4) | <0.001 | 51.6 (54.2) | | 67.9 (64.0) | <0.001 |
| Vegetable (g/day) | 192.7 (105.4) | 320.7 (223.1) | <0.001 | 246.8 (167.9) | 325.3 (204.1) | <0.001 | 241.1 (143.4) | | 308.9 (192.9) | <0.001 |
| Fruit (g/day) | 132.0 (88.2) | 332.5 (282.5) | <0.001 | 238.6 (205.5) | 315.8 (228.4) | <0.001 | 229.2 (196.2) | | 306.2 (220.1) | <0.001 |
| Red Meats (g/day) | 62.8 (50.7) | 56.0 (49.8) | <0.001 | 52.6 (39.3) | 71.1 (60.3) | <0.001 | 63.2 (47.9) | | 56.5 (48.7) | <0.001 |
| White Meats (g/day) | 39.3 (37.5) | 56.4 (52.4) | <0.001 | 39.8 (36.7) | 63.9 (57.5) | <0.001 | 42.0 (38.3) | | 56.4 (50.5) | <0.001 |
| **Nutrient intake** |  |  |  |  |  |  |  | |  |  |
| Fat (g/day) | 58.2 (31.3) | 61.9 (33.1) | <0.001 | 52.6 (25.7) | 73.5 (37.1) | <0.001 | 57.8 (26.5) | | 62.0 (31.9) | <0.001 |
| Carbohydrate (g/day) | 180.2 (74.4) | 233.4 (98.6) | <0.001 | 188.0 (75.9) | 262.1 (93.5) | <0.001 | 203.5 (79.6) | | 228.6 (88.8) | <0.001 |
| Protein (g/day) | 57.3 (26.4) | 68.9 (30.9) | <0.001 | 52.4 (20.7) | 83.6 (32.0) | <0.001 | 60.6 (25.1) | | 68.8 (29.3) | <0.001 |
| Cholesterol (mg/day) | 200.3 (129.5) | 203.0 (132.8) | <0.001 | 169.8 (97.3) | 251.5 (153.2) | <0.001 | 202.4 (119.4) | | 202.1 (126.6) | <0.001 |
| Dietary fiber (g/day) | 13.2 (5.7) | 19.9 (9.5) | <0.001 | 15.3 (7.3) | 21.1 (9.0) | <0.001 | 15.4 (6.6) | | 19.5 (8.7) | <0.001 |
| Sodium (mg/day) | 2,359.0 (1,034.5) | 2,808.7 (1,248.5) | <0.001 | 2,277.7 (922.9) | 3,256.5 (1,286.5) | <0.001 | 2,498.4 (981.0) | | 2,792.6 (1,159.6) | <0.001 |
| ^1^ P value for comparison between quarters of total fat intake, by Kruskal-Wallis rank sum test or pearson's Chi-squared test. | | | | | | |  |  | |  |
| ^2^ Total time of moderate-to-vigorous physical activity per week.  ^3^ Other race/ethnicity = Asian, Pacific Islander or American Indian. | | | | | | |  |  | |  |
