# Supplementary material for: Vitamin intake and colorectal cancer incidence and mortality: evidence from a large prospective cohort
Source: Front Nutr. 2026 Sep 9;13:1877860. doi: 10.3389/fnut.2026.1877860 (PMC13597460; doi:10.3389/fnut.2026.1877860)
Supplement: Supplementary file 2 [file Table_10.docx]

| **Supplementary Table 10.** Sensitivity analyses on the association between vitamin and the mortality of CRC a | | | | | | | | |
| --- | --- | --- | --- | --- | --- | --- | --- | --- |
| Categories | Sample size | Vitamin A | | Vitamin B6 | | Vitamin B12 | |  |
|  |  | Quartile 1 | Quartile 4 | Quartile 1 | Quartile 4 | Quartile 1 | Quartile 4 |  |
| Excluded subjects with extreme  BMIs b | 96608 | 1.00 (reference) | 0.73(0.53, 1.00) | 1.00 (reference) | 0.74(0.55, 0.98) | 1.00 (reference) | 0.78 (0.58, 1.04) | |
| *P-*trend |  |  | 0.015 |  | 0.045 |  | 0.079 | |
| Excluded cases occurred within the first two years of follow-up | 101508 | 1.00 (reference) | 0.72 (0.53,0.98) | 1.00 (reference) | 0.71 (0.53, 0.95) | 1.00 (reference) | 0.79 (0.59, 1.05) | |
| *P-*trend |  |  | 0.015 |  | 0.045 |  | 0.079 | |
| Excluded cases occurred within the first three years of follow-up | 101340 | 1.00 (reference) | 0.71 (0.52, 0.97) | 1.00 (reference) | 0.70 (0.52, 0.93) | 1.00 (reference) | 0.77 (0.57, 1.03) | |
| *P-*trend |  |  | 0.015 |  | 0.045 |  | 0.079 | |
| Excluded cases occurred within the first five years of follow-up | 100235 | 1.00 (reference) | 0.73 (0.53,1.01) | 1.00 (reference) | 0.68 (0.50, 0.93) | 1.00 (reference) | 0.84 (0.62, 1.15) | |
| *P-*trend |  |  | 0.015 |  | 0.045 |  | 0.079 | |
| Additional adjustment on model 3 |  |  |  |  |  |  |  | |
| Healthy Eating Index-2015 | 101692 | 1.00 (reference) | 0.78 (0.58,1.07) | 1.00 (reference) | 0.76 (0.57, 1.01) | 1.00 (reference) | 0.83 (0.63, 1.11) | |
| *P-*trend |  |  | 0.050 |  | 0.093 |  | 0.123 | |
| Intakes of fruit, vegetable and whole grain | 101692 | 1.00 (reference) | 0.71 (0.52,0.98) | 1.00 (reference) | 0.73 (0.55, 0.97) | 1.00 (reference) | 0.81 (0.61, 1.08) | |
| *P-*trend |  |  | 0.011 |  | 0.046 |  | 0.080 | |
| Intakes of, processed and unprocessed red and white meat | 101692 | 1.00 (reference) | 0.75(0.55, 1.02) | 1.00 (reference) | 0.74 (0.56, 0.98) | 1.00 (reference) | 0.81 (0.61, 1.08) | |
| *P-*trend |  |  | 0.021 |  | 0.056 |  | 0.078 | |

a Values are hazard ratios (95% confidence intervals). Hazard ratios were adjusted for the following variables unless otherwise specified: age (years), sex (male, female), and race (non-Hispanic White, non-Hispanic Black, Hispanic, and other race/ethnicity), smoking status [current (>20 cigarettes/day, 10-20 cigarettes/day, <10 cigarettes/day), former (stop smoking >15 years, stop smoking ≤15 years), never], alcohol consumption (g/day), body mass index (kg/m2), aspirin use (yes, no), family history of colorectal cancer (no, yes or possibly), history of colon comorbidity (no, yes), history of diverticulitis or diverticulosis (no, yes), history of colorectal polyp (no, yes), history of diabetes (no, yes), energy intake from diet (kcal/day), physical activity, and educational level, protein(g/day), sodium (mg/day), carbohydrates (g/day), and dietary fiber (g/day).

b Participants who had extreme BMIs (top 2.5% or bottom 2.5% of BMI ).
