# Supplementary material for: Vitamin intake and colorectal cancer incidence and mortality: evidence from a large prospective cohort
Source: Front Nutr. 2026 Sep 9;13:1877860. doi: 10.3389/fnut.2026.1877860 (PMC13597460; doi:10.3389/fnut.2026.1877860)
Supplement: Supplementary file 4 [file Table_12.docx]

**Table 12 The Vitamins Intake Level**

|  | **0%** | **25%** | **50%** | **75%** | **100%** |
| --- | --- | --- | --- | --- | --- |
| **Total vitamin A(mg/day)** | 0.23 | 7.41 | 10.80 | 15.48 | 141.76 |
| **Total vitamin B6(mg/day)** | 0.25 | 1.99 | 3.27 | 4.35 | 72.90 |
| **Total vitamin B12(μg/day)** | 0.16 | 4.32 | 7.96 | 10.12 | 139.78 |
| **Total vitamin C (mg/day)** | 4.93 | 127.74 | 210.18 | 563.15 | 2964.79 |
| **Total vitamin D (μg/day)** | 0.13 | 3.90 | 10.81 | 13.51 | 54.15 |
| **Total vitamin E(mg/day)** | 0.58 | 13.20 | 34.37 | 291.92 | 739.83 |
