# Supplementary material for: Vitamin intake and colorectal cancer incidence and mortality: evidence from a large prospective cohort
Source: Front Nutr. 2026 Sep 9;13:1877860. doi: 10.3389/fnut.2026.1877860 (PMC13597460; doi:10.3389/fnut.2026.1877860)
Supplement: Supplementary file 5 [file Table_2.docx]

**Supplementary Table 2.** Comparison of sociodemographic characteristics between excluded and included populations ^1^

| Sociodemographic characteristics | Excluded population | Included population | Standardized  difference |
| --- | --- | --- | --- |
| Number of participants | 53195 | 101692 |  |
| Age, years | 66.99 ± 5.92 | 65.53 ± 5.73 | 0.25 |
| Sex | | | 0.06 |
| Male | 27227(51.2) | 49451(48.6) |  |
| Female | 25968(48.8) | 52241(51.4) |  |
| Educational degree, | | | |
| Some college or less | 32284 (67.2) | 64706(63.7) | 0.09 |
| College graduate | 7503 (15.6) | 17839 (17.6) |  |
| Postgraduate | 8284 (17.2) | 18943 (18.7) |  |
| Occupation, | | | |
| Working | 18521(38.6) | 40704 (40.2) | 0.04 |
| Retired | 20828 (43.4) | 43680(43.2) |  |
| Other | 8633 (18.0) | 16842 (16.6) |  |
| Smoking status | | | |
| Current | 6661(13.8) | 9392 (9.2) | 0.04 |
| Former | 20871 (43.2) | 43764(43.0) |  |
| Never | 20732 (43.0) | 48536 (47.7) |  |
| Number of cigarettes smoked per day | | | |
| 0 | 20732(42.6) | 48536 (47.7) | 0.05 |
| 1-10 | 7042 (14.5) | 13588 (13.4) |  |
| 11-20 | 9734 (20.0) | 19608 (19.3) |  |
| 21-30 | 5377 (11.1) | 10650(10.5) |  |
| 31-40 | 3181 (6.5) | 5659 (5.6) |  |
| 41-60 | 1703 (3.5) | 2868 (2.8) |  |
| 61-80 | 311 (0.6) | 533 (0.5) |  |
| >80 | 94 (0.2) | 126 (0.1) |  |
| Body mass index ^2^ | 27.39± 5.12 | 27.22 ± 4.79 | 0.02 |
| Family history of colorectal cancer | 4656 (9.7) | 10305 (10.1) | 0.00 |
| History of diabetes | 4731 (9.9) | 6798 (6.7) | 0.06 |
| Aspirin use | 22289(46.6) | 47773(47.2) | 0.03 |
| Trial group | | | |
| Screening group | 25677 (48.3) | 51766 (50.9) | 0.04 |
| Control group | 27518 (51.7) | 49926 (49.1) |  |

^1^ Values are mean ± standard deviation or counts (percentage) as indicated. Standardized difference was calculated using the formula available in the paper by Austin PC. (DOI: 10.1080/00273171.2011.568786).

^2^ Weight (kg)/height (
