# Supplementary material for: Vitamin intake and colorectal cancer incidence and mortality: evidence from a large prospective cohort
Source: Front Nutr. 2026 Sep 9;13:1877860. doi: 10.3389/fnut.2026.1877860 (PMC13597460; doi:10.3389/fnut.2026.1877860)
Supplement: Supplementary file 6 [file Table_3.docx]

**Supplementary Table 3.** Distribution of variables with missing values before and after imputation ^1^

| Covariates | Before imputation | After imputation | Number (%) with missing values |
| --- | --- | --- | --- |
| Educational level | | | |
| Some college or less | 32284 (67.2) | 64706(63.7) | 204 (0.20) |
| College graduate | 7503 (15.6) | 17839 (17.6) |  |
| Postgraduate | 8284 (17.2) | 18943 (18.7) |  |
| Smoking status | | | |
| Current | 9392(9.24) | 9392(9.23) | 20 (0.02) |
| Former | 43744 (43.02) | 43744 (43.01) |  |
| Never | 48536 (47.74) | 48556 (47.76) |  |
| Number of cigarettes smoked per day | | | |
| 0 | 20732(42.6) | 48536 (47.7) | 124(0.12) |
| 1-10 | 7042 (14.5) | 13588 (13.4) |  |
| 11-20 | 9734 (20.0) | 19608 (19.3) |  |
| 21-30 | 5377 (11.1) | 10650(10.5) |  |
| 31-40 | 3181 (6.5) | 5659 (5.6) |  |
| 41-60 | 1703 (3.5) | 2868 (2.8) |  |
| 61-80 | 311 (0.6) | 533 (0.5) |  |
| >80 | 94 (0.2) | 126 (0.1) |  |
| Family history of colorectal cancer | 10305 (11.28) | 10305 (10.1) | 781 (0.77) |
| History of diabetes | 6798(6.71) | 6798(6.68) | 538 (0.55) |
| History of hypertension | 33023(32.6) | 33023(32.5) | 513(0.50) |
| Aspirin use | 47773(47.2) | 47773(47.0) | 444(0.44) |
| Body mass index ^2^ | 27.23± 4.82 | 27.22± 4.79 | 1348(1.33) |
| Physical activity level (min/week) ^3^ | 124.33±122.59 | 122.82±123.26 | 26034 (25.60) |

^1^ n = 101692. Values are mean (standard deviation) or counts (percentage) as indicated.

^2^ Weight (kg)/height (m)^2^.

^3^ Total time of moderate-to-vigorous physical activity per week
