# Supplementary material for: Vitamin intake and colorectal cancer incidence and mortality: evidence from a large prospective cohort
Source: Front Nutr. 2026 Sep 9;13:1877860. doi: 10.3389/fnut.2026.1877860 (PMC13597460; doi:10.3389/fnut.2026.1877860)
Supplement: Supplementary file 9 [file Table_6.docx]

**Supplementary Table 6.** Subgroup analyses on the association between vitamin and the mortality of colorectal cancer^1^

| Subgroup variable | Vitamin A intake and the mortality of colorectal cancer | | Vitamin B6 intake and the mortality of colorectal cancer | | Vitamin B12 intake and the mortality of colorectal cancer | |
| --- | --- | --- | --- | --- | --- | --- |
|  | Quartile 1 | Quartile 4 | Quartile 1 | Quartile 4 | Quartile 1 | Quartile 4 |
| **Age at diet history questionnaire completion (years)** | | | | | | |
| ≥65 | | | | | | |
| HR (95% CI) | 1.00 (reference) | 0.65 (0.45, 0.94) | 1.00 (reference) | 0.75 (0.54, 1.05) | 1.00 (reference) | 0.75 (0.53, 1.04) |
| <65 | | |  |  |  |  |
| HR (95% CI) | 1.00 (reference) | 0.99 (0.58, 1.69) | 1.00 (reference) | 0.69 (0.41, 1.15) | 1.00 (reference) | 1.00 (0.59, 1.69) |
| *P*_interaction_ |  | 0.269 |  | 0.854 |  | 0.574 |
| **Sex** \| \| \| \|; Males \| \| \| \|; HR (95% CI) \| 1.00 (reference) \| 0.58 (0.42, 0.80) \| 1.00 (reference) \| 0.76 (0.51, 1.14); Females \| \| \| \|; HR (95% CI) \| 1.00 (reference) \| 0.93(0.70, 1.24) \| 1.00 (reference) \| 0.75 (0.47, 1.18); *P*_interaction_ \| \| **0.012** \| \| 0.762 | 1.0 (reference)  1.0 (reference) | 0.76 (0.51, 1.14)  0.75 (0.47, 1.18)  0.762 | 1.0 (reference)  1.0 (reference) | 0.71 (0.48, 1.05)  0.73 (0.48, 1.09)  0.821 | 1.0 (reference)  1.0 (reference) | 0.79 (0.54, 1.16)  0.90 (0.58, 1.40)  0.772 |
| **Body mass index (kg/m^2^)** | | | | | | |
| ≥25 | | | | | | |
| HR (95% CI) | 1.00 (reference) | 0.73 (0.51, 1.05) | 1.00 (reference) | 0.69 (0.49, 0.96) | 1.00 (reference) | 0.68 (0.48, 0.95) |
| <25 | | |  | |  | |
| HR (95% CI) | 1.00 (reference) | 0.75 (0.42, 1.34) | 1.00 (reference) | 0.87 (0.51, 1.47) | 1.00 (reference) | 1.31 (0.77, 2.24) |
| *P*_interaction_ |  | 0.766 |  | 0.876 |  | 0.111 |
| **Smoking status** | | |  |  |  |  |
| Current or former smokers stopping smoking ≤15 years | | |  |  |  |  |
| HR (95% CI) | 1.00 (reference) | 0.74 (0.54, 1.02) | 1.00 (reference) | 0.76 (0.57, 1.03) | 1.00 (reference) | 0.82 (0.61, 1.11) |
| Never or former smokers stopping smoking >15 years | | |  |  |  |  |
| HR (95% CI) | 1.00 (reference) | 0.73 (0.24, 2.29) | 1.00 (reference) | 0.39 (0.13, 1.18) | 1.00 (reference) | 0.74 (0.30, 1.79) |
| *P*_interaction_ |  | 0.219 |  | 0.093 |  | 0.210 |
| **Alcohol consumption** | | |  |  |  |  |
| ≥ median | | |  |  |  |  |
| HR (95% CI) | 1.00 (reference) | 0.66 (0.43, 1.01) | 1.00 (reference) | 0.69 (0.46, 1.03) | 1.00 (reference) | 0.70 (0.47, 1.04) |
| < median |  |  |  |  |  |  |
| HR (95% CI) | 1.00 (reference) | 0.84 (0.55, 1.03) | 1.00 (reference) | 0.77 (0.52, 1.14) | 1.00 (reference) | 1.01 (0.68, 1.52) |
| *P*_interaction_ |  | 0.462 |  | 0.704 |  | 0.133 |
| **Aspirin use** | | |  |  |  |  |
| Yes | | |  |  |  |  |
| HR (95% CI) | 1.00 (reference) | 0.62 (0.39, 0.99) | 1.00 (reference) | 0.71 (0.46, 1.09) | 1.00 (reference) | 0.73 (0.48, 1.11) |
| No | | |  |  |  |  |
| HR (95% CI) | 1.00 (reference) | 0.86 (0.57, 1.28) | 1.00 (reference) | 0.77 (0.53, 1.12) | 1.00 (reference) | 0.91 (0.62, 1.32) |
| *P*_interaction_ |  | 0.750 |  | 0.832 |  | 0.749 |
| **Trial group** | | |  |  |  |  |
| Screening group | | |  |  |  |  |
| HR (95% CI) | 1.00 (reference) | 0.77 (0.48, 1.25) | 1.00 (reference) | 0.73 (0.47, 1.14) | 1.00 (reference) | 0.84 (0.54, 1.32) |
| Control group | | |  |  |  |  |
| HR (95% CI) | 1.00 (reference) | 0.71 (0.48, 1.05) | 1.00 (reference) | 0.73 (0.51, 1.04) | 1.00 (reference) | 0.79 (0.55, 1.13) |
| *P*_interaction_ |  | 0.695 |  | 0.782 |  | 0.621 |

^1^ n = 101692. Hazard ratios and 95% confidence intervals were calculated using the Cox proportional hazards regression model. HRs were adjusted for age at diet history questionnaire completion (years), sex (males, females), race (non-Hispanic White, non-Hispanic Black, Hispanic, and other race/ethnicity), body mass index (kg/m^2^), alcohol consumption (g/d), smoking status [current (>20 cigarettes/day, 11-20 cigarettes/day, ≤10 cigarettes/day), former (stop smoking >15 years, stop smoking ≤15 years), never], family history of colorectal cancer (no, yes or possibly), history of colon comorbidity (no, yes), history of diverticulitis or diverticulosis (no, yes), history of colorectal polyp (no, yes), history of diabetes (no, yes). In subgroup analyses stratified by sex, aspirin use, and smoking status, hazard ratios were not adjusted for the stratification factor. The *P*_interaction_ was calculated by comparing models with and without interaction terms. CI, confidence interval; HR, hazard ratio.
